# The Association between Blood Concentrations of PCDD/DFs, DL-PCBs and the Risk of Type 2 Diabetes Mellitus and Thyroid Cancer in South Korea

**DOI:** 10.3390/ijerph19148745

**Published:** 2022-07-18

**Authors:** SuHyun Lee, YoungWook Lim, YounSeok Kang, KeumJi Jung, SunHa Jee

**Affiliations:** 1 Department of Epidemiology and Health Promotion, Institute for Health Promotion, Graduate School of Public Health, Yonsei University, Seoul 03722, Korea; szhzzn3@yuhs.ac (S.L.); jsunha@yuhs.ac (S.J.); 2 Department of Public Health, Graduate School, Yonsei University, Seoul 03722, Korea; 3 Institute for Environmental Research, Yonsei University College of Medicine, Seoul 03722, Korea; envlim@yuhs.ac; 4 Environment Testing Division, Eurofins Korea Ltd., Gunpo 15849, Korea; michaelkang@eurofins.com

**Keywords:** PCDD, PCDFs, dioxin-like PCBs, type 2 diabetes mellitus, thyroid cancer, epidemiology

## Abstract

Background and Objectives: Epidemiological studies have inconsistently shown an association between dioxin and risk of type 2 diabetes mellitus (T2DM) and cancer. This study aims to examine the effects of blood concentration of dioxin-like polychlorinated biphenyls (DL-PCBs) and polychlorinated dibenzo-p-dioxins and dibenzofurans (PCDD/DFs) on T2DM and thyroid cancer. Methods: We conducted a nested case–control study within the Korean cancer prevention study-II (KCPS-II) consisting of 15 thyroid cancer cases, 30 T2DM cases, and 55 controls. A total of 500 samples were used in 100 pooling samples. An average value of a pooled sample was calculated weighted by the blood volume of each sample. Results: The study population included 100 participants from the KCPS-II (median (IQR) baseline age, 54.06 [21.04] years; 48 women). The toxic equivalents of PCDD/DFs showed a significant positive association with T2DM and thyroid cancer, after adjustments for potential confounders (T2DM ORs  =  1.23; 95% CI  =  1.05–1.43; thyroid cancer ORs  =  1.34; 95% CI  =  1.12–1.61). Conclusion: In this study, both T2DM and thyroid cancer were associated with the blood concentrations of PCDD/DFs. The association between PCDD/DFs and T2D was found among women but not among men. Our findings suggest that further biochemical in vivo research and epidemiologic studies are needed to clarify the association between dioxins concentrations and diseases.

## 1. Introduction

Dioxins are a group of chemical compounds consisting of 75 polychlorinated dibenzo-p-dioxin (PCDD) and 135 polychlorinated dibenzofurans (PCDFs) that are formed by waste incineration processes, automobile emissions, and cigarette smoking [1,2,3,4]. Polychlorinated dibenzo-p-dioxins, dibenzofurans (PCDD/DFs), and dioxin-like polychlorinated biphenyls (DL-PCBs) are persistent environmental pollutants (POPs), which are compounds that accumulate in the environment and human body [5,6].

An epidemiological investigation of a group exposed to a relatively high concentration of dioxins due to an accident or occupation showed a significant relationship between blood dioxins concentration and the onset of T2DM or death from T2DM [7,8,9,10,11,12,13,14]. However, studies on the relationship between dioxins concentration in blood and T2DM identified in groups exposed to relatively high concentrations of dioxins due to accidents or occupations have yielded inconsistent results [10,12]. In addition, these studies used the half-life of dioxins in the body, which may reduce the accuracy of the exposure assessment; thus, caution is needed when interpreting the results. In recent years, there have been studies on the association between low dioxins concentration, PCBs exposure, and T2DM in general environments, but few studies were conducted among Asians [15,16]. 

In 1997, the International Agency for Research on Cancer classified TCDD as a human carcinogen [17]. Among PCDD, 2,3,7,8-tetrachlorodibenzo-p-dioxin (TCDD) is the most toxic to the aryl hydrocarbon-receptor, and molecular studies have shown that TCDD is a strong carcinogen that can disrupt various endocrine pathways in animals and humans [5,18]. However, the relationship between TCDD and cancer incidence or mortality has been inconsistent in epidemiological studies [7,11,12,14,19,20,21,22,23,24,25,26,27,28,29,30,31,32]. Previous meta-analysis showed that the external exposure and blood level of TCDD were both significantly associated with all cancer mortality, but the meta-analysis did not include studies of the relationship with thyroid cancer [33]. The majority of the dioxins studies are animal-based experiments and few studies have been conducted with respect to human research, which may be due to the difficulties in obtaining about 4 mL of whole blood to measure dioxins in human blood [34]. In addition, it takes a long period of time to conduct a prospective cohort study of the association between exposure to dioxins and disease development. Therefore, we pooled the amount of blood needed for dioxin analysis using the pooling method in large-scale cohort data and investigated whether the blood concentration of DL-PCBs and PCDD/DFs is associated with T2DM and thyroid cancer in a Korean population.

## 2. Methods

### 2.1. Study Population

This study design was a nested case–control study of 500 Korean adults (men: 263, women: 237) selected from the Korean Cancer Prevention Study-II (KCPS-II) (Supplementary Figure S1). The KCPS-II included 156,704 adults aged 20 to 84 who visited 18 health promotion centers nationwide from April 2004 to December 2013. A detailed description of the KCPS-II study design was published elsewhere [35]. 

In this study, the baseline period for the blood sample collection was determined from January 2004 to December 2004. Due to the 4 mL human blood requirement for dioxins tests [36], a blood sample was produced by combining 0.3 mL to 1.0 mL of the individual serum sample of five to nine participants. The subjects with disease samples were pooled with considerations for sex and age, and the control-group sample was pooled with consideration of the sex, age and body mass index (BMI) of the disease group. A total of 500 samples were used in 100 pooling samples consisting of 30 cases of T2DM, 15 cases of thyroid cancer, and 55 for the control group (Supplementary Figure S2).

### 2.2. Measurements

#### 2.2.1. Data Source

After 12 h of fasting, serum and whole blood were collected from each participant and placed into storage at −70 °C for future studies. These samples were used for PCDD/DFs, DL-DCBs measurements and other clinical chemistry parameters, such as fasting blood glucose, total cholesterol, triglyceride, HDL-C and LDL-C. 

In compliance with the protocols of the Korean Organization of Laboratory Quality Management, the quality control of the clinical chemistry laboratory was maintained.

The incidence of thyroid cancer was ascertained from the registry of the National Cancer Center using the 10th Amendment to the International Classification of Diseases (ICD-10) code (ICD-10 C73). The incidence of T2DM was identified from the National Health Insurance System (NHIS).

#### 2.2.2. Analysis of Dioxins

The analysis and quality control of 2,3,7,8-substituted PCDD/DFs and DL-PCBs in the pooled serum samples were performed with a slight modification of the Center for Disease Control and Prevention [37]. Before solid phase extraction, pooled serum samples were spiked 13C-labeled 2,3,7,8-substituted PCDD/DFs and DL-PCBs and underwent homogenization. A series of 20 samples was processed on manifolds. Formic acid and pure water were added to samples prior to extraction. SPE-C18 cartridges (octadecyl, 2 g) were pre-conditioned using methanol and water. Each cartridge was dried and eluted with 15 mL of hexane, followed by concentrating the elute to 1 mL. The eluate was applied to a multilayer silica gel column (44% sulfuric acid and 10% AgNo_3_ silica gel) and then eluted with 20 mL of hexane. The final evaporation using a nitrogen concentrator (Eyela MGS 3100) was performed after the addition of nonane as a keeper. The quantification and identification of 2,3,7,8-substituted PCDD/DFs congeners and DL-PCBs were performed by high-resolution gas chromatography (HRGC) (Thermo scientific Trace 1310)/high-resolution mass spectrometry (HRMS) (Thermo Scientific DFS). The HRMS operated in the electron-impact mode and in the selected ion-monitoring mode at a resolution of R > 10,000 (10% valley). Separation was achieved using an HRGC instrument equipped with a DB-5MS (Agilent Technologies; 60 m length, 0.32 mm i.d., 0.25 µm film thickness) capillary column with a splitless and solvent-cut mode. The column ovens for DB5-MS were programmed from an initial temperature of 160 °C to a final temperature of 310 °C (total running time 60 min). Before quantitative analysis, 13C-labeled 1,2,3,4-TeCDD, 1,2,3,7,8,9-HxCDD, 3,3′,4,5′-TetraCB, 2,3,3′,5,5′-pentaCB and 2,3,3′,4,5,5′-hexaCB as internal standards were added for the estimation of recovery. The mean recoveries of the spiked 13C-labeled 2,3,7,8-substituted PCDD/DFs and DL-PCBs in the entire analytical procedures were 75 ± 11% and 80 ± 25%, respectively. The levels were expressed in 2,3,7,8-TeCDD toxic equivalents using calculations of World Health Organization Toxic Equivalent Factors (WHO-TEFs) for PCDD/DFs and DL-PCBs.

### 2.3. Statistical Analysis

As POPs are mainly carried in the lipid portion of the blood, epidemiological studies have used lipid-adjusted concentrations (ng/g lipid) [38]. Concentrations adjusted for lipids (ng/g lipids) were determined using the formula proposed by Bernert et al. (2007) [39]. 

The continuous variable of a pooled sample of 500 samples was calculated as an average value weighed by the blood volume of each sample. The average value was calculated by the PROC SURVEYSMEANS statement in SAS 9.4 and the equation below was used.

(1) $$\mathrm{Average}\;\mathrm{value}\;\mathrm{considering}\;\mathrm{the}\;\mathrm{weight}\;\mathrm{of}\;\mathrm{blood}\;\mathrm{volume}\;\;=\sum \left(blood\;volume\times value\right)\;/\sum \left(blood\;volume\right)$$

We reported descriptive statistics for continuous variables using medians (±interquartile range; IQR) and categorical variables using proportions. We conducted the Kruskal–Wallis test to analyze between-group differences. Logistic regression analyses were conducted to estimate the odds ratio (OR) for the association between PCDD/DFs, DL-PCBs concentrations and thyroid cancer and T2DM. We performed sensitivity analyses in which multivariable logistic regressions were adjusted for predefined baseline covariates. *p*-values ≤ 0.05 in the two-tailed test were considered significant. Statistical analyses were performed with the use of SAS software, version 9.4 (SAS Institute, Cary, NC, USA), and R software, version 4.1.2 (R Foundation for Statistical Computing).

## 3. Results

### 3.1. Study Population and PCDD/DFs, DL-PCBs, and Total Dioxins in Blood

The characteristics of the study population are described in Table 1. The numbers of the non-disease group, T2DM and thyroid cancer were 55, 30, and 15, respectively. The participants were distributed almost equally according to sex, age and BMI by design. The proportions of men and women were 48% and 52%, respectively. The median value of age was 54.06 years (IQR = 21.04), and the median value of BMI was 24.28 kg/m^2^ (IQR = 2.15). 

**Figure 1:**
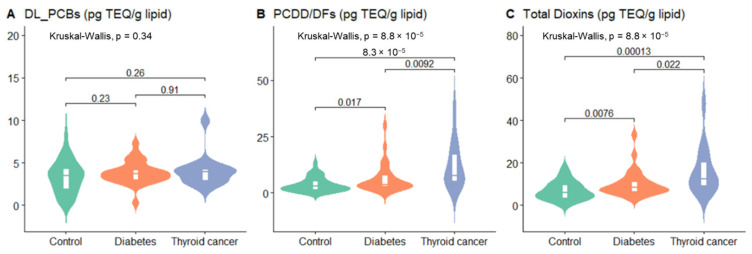
Serum concentrations for PCDD/DFs, DL-PCBs of the study group. Violin plots show differences in blood of concentrations for DL_PCBs (**A**), PCDD/DFs (**B**), total dioxins (**C**) at the controls group (*n* = 55), type 2 diabetes (*n* = 30) or thyroid cancer (*n* = 15). The line in the white box represents the median. The width of the shape represents blood concentration density, and the length illustrates the range of the blood concentration.

Figure 1 shows the blood TEQ concentration of PCDD/DFs, DL-PCBs and total dioxins. For the TEQ of DL_PCBs, the difference in exposure levels between groups was not statistically significant (*p*-value ≤ 0.34). The median of the PCDD/DFs and total dioxins material the was highest in the thyroid cancer group, and the difference in exposure levels between groups was statistically significant (both *p*-value ≤ 0.01).

### 3.2. Association between Dioxins in Blood and T2DM

**Figure 2:**
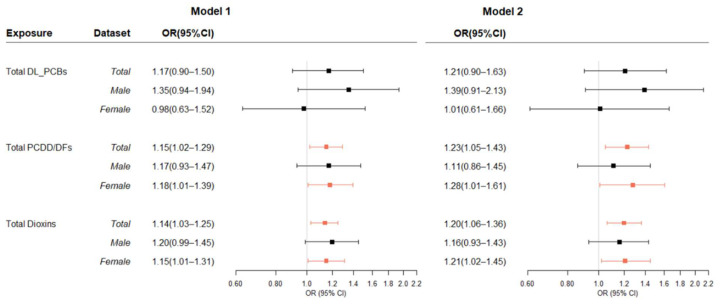
Odds Ratios of blood concentrations of PCDD/DFs, DL-PCBs (pgTEQ/g_lipid) and Type 2 Diabetes mellitus. OR, odds ratio; CI, confidence interval; Model 1: Adjusted for age and sex; Model 2: Adjusted for the model 1 variables, body mass index, systolic blood pressure and high-density lipoprotein. Marked red based on statistically significant results.

The multiple-adjusted associations between blood levels of dioxins and T2DM are presented in Figure 2. The PCDD/DFs TEQ and total dioxins, but not DL-PCBs, showed significant associations with T2DM. The age and sex-adjusted ORs of T2DM and total dioxins were 1.14 (95% CI = 1.03–1.25). Stratifying analyses by sex showed positive association, but they were not statistically significant in men (men ORs = 1.20; 95% CI = 0.99–1.45, women ORs = 1.15; 95% CI = 1.01–1.31). 

To assess the confounding effect by BMI, systolic blood pressure and high-density lipoprotein, an analysis for T2DM was performed by additionally adjusting for the effects of these variables. In the adjusted models that include these variables (Model 2), the OR for total dioxins was 1.20 (95% CI = 1.06–1.36). The results of Model 2 also showed statistically significant results only in the women group (men ORs = 1.16; 95% CI = 0.93–1.43, women ORs = 1.21; 95% CI = 1.02–1.45). Specifically, a 1-SD increase in 2378-TCDF levels was associated with a 71% increased risk of T2DM (ORs = 1.71; 95% CI = 1.0–2.91) (Supplementary Table S2).

### 3.3. Association between Dioxins in Blood and Thyroid Cancer

**Figure 3:**
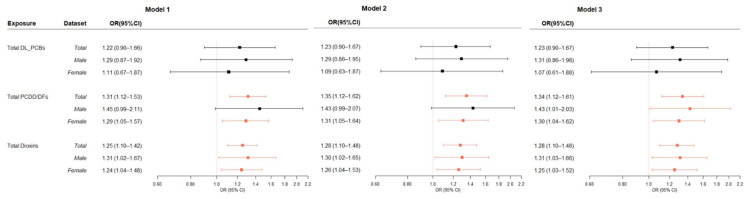
Odds Ratios of blood concentrations of PCDD/DFs, DL-PCBs (pgTEQ/g_lipid) and Thyroid Cancer. OR, odds ratio; CI, confidence interval. Model 1: Adjusted for age and sex; Model 2: Adjusted for the model 1 variables and body mass index; Model 3: Adjusted for the model 2 variables and thyroid stimulating hormone. Marked red based on statistically significant results.

The multiple-adjusted associations between blood levels of dioxins and thyroid cancer are presented in Figure 3. The TEQ of PCDD/DFs and total dioxins, but not DL-PCBs, showed significant associations with thyroid cancer. The age- and sex-adjusted ORs of thyroid cancer of total dioxins were 1.25 (95% CI = 1.10–1.42). Both men and women subgroup analyses showed positive associations (men ORs = 1.31; 95% CI = 1.02–1.67), women ORs = 1.24; 95% CI = 1.04–1.48). To assess the possibility of confounding by TSH serum levels or BMI, an analysis for thyroid cancer was performed by additionally adjusting for the effects of these variables. In the model additionally adjusted for BMI (Model 2) or TSH serum levels (Model 3), the OR for total dioxins was 1.28 for both models (both models 95% CI = 1.10–1.48). The DL-PCB TEQ did not show a significant association with thyroid cancer. However, the 1-SD increase at 4CB-77 levels was associated with an increase in risk of thyroid cancer (OR = 1.92; 95% CI = 1.02–3.64) (Supplementary Table S3).

## 4. Discussion

This study measured DL_PCB and PCDD/DFs using pooled serum from a general population. PCDD/DFs in blood showed a significant positive association with T2DM and thyroid cancer development, but no significant association with DL_PCB was observed.

In 2016, the International Agency for Research on Cancer (IARC) upgraded the classification of PCBs to Category 1 carcinogenic to humans from the previous Category 2A classification on the basis of observing sufficient evidence for carcinogenicity in humans and animals [17,40]. However, no evidence of a relationship between PCB exposure and the risk of malignant melanoma has been identified in the latest meta-analysis [41], and the results of the epidemiological studies on PCB exposure and cancer risk were inconsistent for other cancers [42]. Our findings of non-significant associations of DL_PCBs with cancer are consistent with prior studies [41,42]. 

Although previous studies have found an association between PCB levels and T2DM in women, our results are inconsistent with the reports from other studies, suggesting that PCBs are positively associated with T2DM in women [43,44]. However, the PCBs used in previous studies are a combination of DL-PCBs and non-dioxin-PCBs. In this study, however, we only included DL-PCBs for PCBs.

To the best of our knowledge, no case–control studies have yet been conducted to establish an association between dioxins and thyroid cancer. A recent in vitro study using immortal mouse cells has shown that TCDD exposure regulates the script of an endothelial carcinogen network thought to affect thyroid carcinoma [45]. TCDD can interfere with the activity and metabolism of thyroid hormones through various processes, including binding to the protein transport of thyroid hormones [46], direct damage to the thyroid gland, and the activation of thyroid-metabolizing enzymes [47]. Previous epidemiological studies have found significantly increasing trends in mean TSH with TCDD category [48]. It has been suggested that having a high TSH level within the normal range is an independent risk factor for differentiated thyroid cancer, and this may contribute to the initiation of thyroid carcinogenesis [49,50] These mechanisms can support the association between blood PCDD/DFs and thyroid cancer risks identified in our study.

Dioxins have been identified as endocrine disruptors of the environment, but epidemiology studies of their effect on diabetes found inconsistent results [8,9,32,51]. In particular, this association was found in women and not in men [7,44] which is similar to our results. Several assumptions can explain this gender difference. First, men have lower levels of exposure and a higher prevalence of smoking, which stimulates the aryl hydrocarbon receptor related to the increased excretion of PCBs [52]. Second, women have a higher proportion of fat, resulting in these lipophilic compounds being stored longer. Third, women have higher estrogen levels and PCDFs. Certain PCBs can cause the gene expression of CYP1A1 and CYP1B1 [53,54], which catalyze estradiol A-ring hydroxylation to form 4-hydroxyl estradiol of catechol estrogen, which can produce free radicals. It is understood that free radicals induce elevated oxidative stress related to diabetes [53].

Nonetheless, there are some limitations in our study. First, we were unable to control for potential confounding variables such as exercise habits, food consumption, alcohol status, smoking status, and socioeconomic status. In this study, several blood samples were used to make pooled samples. In the case of a continuous variable, the mean value of the characteristics constituting the sample was used. Moreover, categorical variables were not included in this study. However, we used BMI, which is strongly associated with physical activity patterns, waist circumference, and dietary consumption and may, thus, be considered a proxy indicator for such variables. Second, as an exposure measure, we used a one-time dioxins level measurement in the blood and did not have accumulated exposure dose information. However, we measured PCDD/DFs and DL-PCBs concentrations directly within the Korean population to collect exposure data. Moreover, since the half-life of PCDD/DFs in the serum can last for seven years or longer [51,55] and assuming that the causes of environmental exposure remained constant over time, we can conclude that the dioxins level in a given participant has remained similar over the years. Third, in this study, dioxin levels were determined using a pooled sample. Data were generated using a statistical model after pooling individuals with similar demographic characteristics, but this may not explain the variability and uncertainty among pooled individuals. The method of pooling individual serum samples, on the other hand, has the advantages of improved detection rates and low cost. According to a 2017 paper, the United States now biomonitors dioxin-like compounds in a pooled sample [56]. Furthermore, this study suggested that comparing dioxins, furans and PCBs within the same population is beneficial. We expect good internal validity because our study is based on large-scale prospective cohort study data from the Korean population. This study also has several strengths. To the best of our knowledge, this is the first study investigating the association between blood concentration of dioxin and health outcomes in general populations with low dose exposures. Moreover, the analysis showed the possibility of studying dioxins that need a large amount of blood for detection using a pooled sample. Further studies on the health impact mechanism of dioxin are warranted.

## 5. Conclusions

To our knowledge, this is the first study that shows the association between PCDD/DFs, DL-PCBs serum levels and T2DM and thyroid cancer risk in the Korean population. In this study, both T2DM and thyroid cancer appear to have an association with PCDD/DFs serum levels. Our findings suggest that further biochemical in vivo research and epidemiologic studies are needed to clarify the nature of the association between dioxins concentration and diseases.

## Figures and Tables

**Table 1:** Baseline characteristics of 500 individuals who constructed 100 pooled samples in the KCPS-II.

Characteristic	Normal Range	Overall	Control (*n* = 55)	Type 2 Diabetes (*n* = 30)	Thyroid Cancer (*n* = 15)	*p*-Value
Sex			*n* (%)			0.795
Male		48 (48.00)	27 (49.09)	15 (50.00)	6 (40.00)	
Female		52 (52.00)	28 (50.91)	15 (50.00)	9 (60.00)	
			Median (IQR)			
Age, years		54.06 (21.04)	54.25 (20.57)	54.04 (22.14)	52.60 (19.20)	0.813
BMI, kg/m^2^	25–29.9	24.28 (2.15)	24.31 (1.94)	24.36 (2.21)	24.20 (1.83)	0.619
FBS, mg/dL	70–100	92.88 (14.23)	89.42 (6.73)	135.73 (40.13)	92.88 (7.59)	≤0.001
HDL-C, mg/dL	40 or higher	52.89 (9.57)	55.20 (12.23)	51.56 (6.70)	50.00 (5.23)	0.004
LDL-C, mg/dL	Less than 110	117.32 (26.32)	112.35 (27.29)	121.61 (22.17)	115.51 (18.99)	0.046
SBP, mmHg	Less than 120	123.71 (14.35)	121.81 (10.95)	129.50 (12.44)	116.25 (16.32)	0.005
TG, mg/dL	Less than 150	135.95 (66.38)	131.56 (61.10)	163.28 (67.74)	125.05 (45.32)	0.009
TSH, uIU/mL	0.35–5.5	1.66 (0.83)	1.66 (0.82)	1.64 (0.79)	1.69 (0.92)	0.659

IQR, interquartile range; BMI, body mass index; SBP, systolic blood pressure; FBS, fasting blood sugar; HDL-C, high-density lipoprotein-cholesterol; LDL-C, low-density lipoprotein-cholesterol; TG, triglyceride; TSH, thyroid stimulation hormone; kg/m^2^, kilogram per square meter; mg/dL, milligrams per deciliter; mmHg, millimeters of mercury; uIU/mL, micro-international units per milliliter; *p* values from Kruskal–Wallis test and all variables are calculated by weighted blood volume.

## Data Availability

Due to the nature of this research, participants of this study did not agree for their data to be shared publicly, so supporting data are not available.

## Supplementary Materials

The following are available online at https://www.mdpi.com/article/10.3390/ijerph19148745/s1, Table S1: Serum concentrations for homologue of PCDD/DFs, DL-PCBs of the study group. Table S2: Odds Ratios of blood concentrations for homologue of PCDD/DFs, DL-PCBs (per 1 SD) and Type 2 Diabetes mellitus. Table S3: Odds Ratios of blood concentrations for homologue of PCDD/DFs, DL-PCBs (per 1 SD) and Thyroid Cancer. Figure S1: Flow Chart: Sample for the Analysis, KCPS-II. Figure S2: Schematic diagram showing different groups of blood samples tested for blood concentrations of PCDD/DFs, DL-PCBs (pgTEQ/g_lipid).

## References

[1] Andersson P., McGuire J., Rubio C., Gradin K., Whitelaw M.L., Pettersson S., Hanberg A., Poellinger L. (2002). A constitutively active dioxin/aryl hydrocarbon receptor induces stomach tumors. Proc. Natl. Acad. Sci. USA.

[2] Birnbaum L.S., Couture L.A. (1988). Disposition of octachlorodibenzo-p-dioxin (OCDD) in male rats. Toxicol. Appl. Pharm..

[3] Geyer H., Scheunert I., Korte F. (1986). Bioconcentration potential of organic environmental chemicals in humans. Regul. Toxicol. Pharm..

[4] Lakshmanan M.R., Campbell B.S., Chirtel S.J., Ekarohita N., Ezekiel M. (1986). Studies on the mechanism of absorption and distribution of 2,3,7,8-tetrachlorodibenzo-p-dioxin in the rat. J. Pharm. Exp. Ther..

[5] Birnbaum L.S. (1994). The mechanism of dioxin toxicity: Relationship to risk assessment. Environ. Health Perspect..

[6] WHO (2020). Persistent Organic Pollutants (POPs). Retrieved 2020.11. https://www.who.int/foodsafety/areas_work/chemical-risks/pops/en/.

[7] Bertazzi P.A., Consonni D., Bachetti S., Rubagotti M., Baccarelli A., Zocchetti C., Pesatori A.C. (2001). Health effects of dioxin exposure: A 20-year mortality study. Am. J. Epidemiol..

[8] Calvert G.M., Sweeney M.H., Deddens J., Wall D.K. (1999). Evaluation of diabetes mellitus, serum glucose, and thyroid function among United States workers exposed to 2,3,7,8-tetrachlorodibenzo-p-dioxin. Occup. Environ. Med..

[9] Henriksen G.L., Ketchum N.S., Michalek J.E., Swaby J.A. (1997). Serum dioxin and diabetes mellitus in veterans of Operation Ranch Hand. Epidemiology.

[10] Steenland K., Calvert G., Ketchum N., Michalek J. (2001). Dioxin and diabetes mellitus: An analysis of the combined NIOSH and Ranch Hand data. Occup Environ. Med..

[11] Bertazzi P.A., Bernucci I., Brambilla G., Consonni D., Pesatori A.C. (1998). The Seveso studies on early and long-term effects of dioxin exposure: A review. Environ. Health Perspect.

[12] Steenland K., Piacitelli L., Deddens J., Fingerhut M., Chang L.I. (1999). Cancer, Heart Disease, and Diabetes in Workers Exposed to 2,3,7,8-Tetrachlorodibenzo-p-dioxin. JNCI J. Natl. Cancer Inst..

[13] Ukropec J., Radikova Z., Huckova M., Koska J., Kocan A., Sebokova E., Drobna B., Trnovec T., Susienkova K., Labudova V. (2010). High prevalence of prediabetes and diabetes in a population exposed to high levels of an organochlorine cocktail. Diabetologia.

[14] Vena J., Boffetta P., Becher H., Benn T., Bueno-de-Mesquita H.B., Coggon D., Colin D., Flesch-Janys D., Green L., Kauppinen T. (1998). Exposure to dioxin and nonneoplastic mortality in the expanded IARC international cohort study of phenoxy herbicide and chlorophenol production workers and sprayers. Environ. Health Perspect..

[15] Hoyeck M.P., Blair H., Ibrahim M., Solanki S., Elsawy M., Prakash A., Rick K.R.C., Matteo G., O’Dwyer S., Bruin J.E. (2020). Long-term metabolic consequences of acute dioxin exposure differ between male and female mice. Sci. Rep..

[16] Taylor K.W., Novak R.F., Anderson H.A., Birnbaum L.S., Blystone C., Devito M., Jacobs D., Köhrle J., Lee D.-H., Rylander L. (2013). Evaluation of the Association between Persistent Organic Pollutants (POPs) and Diabetes in Epidemiological Studies: A National Toxicology Program Workshop Review. Environ. Health Perspect..

[17] (2016). Polychlorinated Biphenyls and Polybrominated Biphenyls: IARC Monographs on the Evaluation of Carcinogenic Risks to Humans: 107. https://monographs.iarc.who.int/wp-content/uploads/2018/08/mono107.pdf.

[18] Birnbaum L.S. (1995). Developmental effects of dioxins and related endocrine disrupting chemicals. Toxicol. Lett..

[19] Cole P., Trichopoulos D., Pastides H., Starr T., Mandel J.S. (2003). Dioxin and cancer: A critical review. Regul. Toxicol. Pharmacol..

[20] Collins J.J., Strauss M.E., Levinskas G.J., Conner P.R. (1993). The mortality experience of workers exposed to 2,3,7,8-tetrachlorodibenzo-p-dioxin in a trichlorophenol process accident. Epidemiology.

[21] Kogevinas M., Becher H., Benn T., Bertazzi P.A., Boffetta P., Bueno-de-Mesquita H.B., Coggon D., Colin D., Flesch-Janys D., Fingerhut M. (1997). Cancer mortality in workers exposed to phenoxy herbicides, chlorophenols, and dioxins. An expanded and updated international cohort study. Am. J. Epidemiol..

[22] Kogevinas M., Saracci R., Winkelmann R., Johnson E.S., Bertazzi P.A., Bueno de Mesquita B.H., Kauppinen T., Littorin M., Lynge E., Neuberger M. (1993). Cancer incidence and mortality in women occupationally exposed to chlorophenoxy herbicides, chlorophenols, and dioxins. Cancer Causes Control..

[23] Manz A., Flesch-Janys D., Waltsgott H., Berger J., Nagel S., Dwyer J.H. (1991). Cancer mortality among workers in chemical plant contaminated with dioxin. Lancet.

[24] Pavuk M., Cerhan J.R., Lynch C.F., Schecter A., Petrik J., Chovancova J., Kocan A. (2004). Environmental exposure to PCBs and cancer incidence in eastern Slovakia. Chemosphere.

[25] Pesatori A.C., Consonni D., Rubagotti M., Grillo P., Bertazzi P.A. (2009). Cancer incidence in the population exposed to dioxin after the “Seveso accident”: Twenty years of follow-up. Environ. Health.

[26] Revich B., Aksel E., Ushakova T., Ivanova I., Zhuchenko N., Klyuev N., Brodsky B., Sotskov Y. (2001). Dioxin exposure and public health in Chapaevsk, Russia. Chemosphere.

[27] Reynolds P., Hurley S.E., Petreas M., Goldberg D.E., Smith D., Gilliss D., Mahoney M.E., Jeffrey S.S. (2005). Adipose levels of dioxins and risk of breast cancer. Cancer Causes Control..

[28] Tuomisto J.T., Pekkanen J., Kiviranta H., Tukiainen E., Vartiainen T., Tuomisto J. (2004). Soft-tissue sarcoma and dioxin: A case-control study. Int. J. Cancer.

[29] Villeneuve S., Cyr D., Lynge E., Orsi L., Sabroe S., Merletti F., Gorini G., Morales-Suarez-Varela M., Ahrens W., Baumgardt-Elms C. (2010). Occupation and occupational exposure to endocrine disrupting chemicals in male breast cancer: A case-control study in Europe. Occup. Environ. Med..

[30] Warner M., Eskenazi B., Mocarelli P., Gerthoux P.M., Samuels S., Needham L., Patterson D., Brambilla P. (2002). Serum dioxin concentrations and breast cancer risk in the Seveso Women’s Health Study. Environ. Health Perspect..

[31] Zambon P., Ricci P., Bovo E., Casula A., Gattolin M., Fiore A.R., Chiosi F., Guzzinati S. (2007). Sarcoma risk and dioxin emissions from incinerators and industrial plants: A population-based case-control study (Italy). Environ. Health.

[32] Zober A., Messerer P., Huber P. (1990). Thirty-four-year mortality follow-up of BASF employees exposed to 2,3,7,8-TCDD after the 1953 accident. Int. Arch. Occup. Environ. Health.

[33] Xu J., Ye Y., Huang F., Chen H., Wu H., Huang J., Hu J., Xia D., Wu Y. (2016). Association between dioxin and cancer incidence and mortality: A meta-analysis. Sci. Rep..

[34] Ball M. (2012). Dioxins, Furans and WHO PCB in Whole Blood [Biomonitoring Methods, 2003].

[35] Jee Y.H., Emberson J., Jung K.J., Lee S.J., Lee S., Back J.H., Hong S., Kimm H., Sherliker P., Jee S.H. (2018). Cohort Profile: The Korean Cancer Prevention Study-II (KCPS-II) Biobank. Int. J. Epidemiol..

[36] Srogi K. (2008). Levels and congener distributions of PCDDs, PCDFs and dioxin-like PCBs in environmental and human samples: A review. Environ. Chem. Lett..

[37] CDC (2016). Laboratory Procedure Manual Method 6501.04, Centers for Disease Control and Prevention. https://wwwn.cdc.gov/nchs/data/nhanes/2009-2010/labmethods/DOXPOL_F_MET.pdf.

[38] Lee D.-H., Lind P.M., Jacobs D.R., Salihovic S., Van Bavel B., Lind L. (2011). Polychlorinated Biphenyls and Organochlorine Pesticides in Plasma Predict Development of Type 2 Diabetes in the Elderly. Diabetes Care.

[39] Bernert J.T., Turner W.E., Patterson D.G., Needham L.L. (2007). Calculation of serum “total lipid” concentrations for the adjustment of persistent organohalogen toxicant measurements in human samples. Chemosphere.

[40] Lauby-Secretan B., Loomis D., Grosse Y., Ghissassi F.E., Bouvard V., Benbrahim-Tallaa L., Guha N., Baan R., Mattock H., Straif K. (2013). Carcinogenicity of polychlorinated biphenyls and polybrominated biphenyls. Lancet Oncol..

[41] Boffetta P., Catalani S., Tomasi C., Pira E., Apostoli P. (2018). Occupational exposure to polychlorinated biphenyls and risk of cutaneous melanoma: A meta-analysis. Eur. J. Cancer Prev..

[42] Zani C., Toninelli G., Filisetti B., Donato F. (2013). Polychlorinated biphenyls and cancer: An epidemiological assessment. J. Environ. Sci. Health C Environ. Carcinog. Ecotoxicol. Rev..

[43] Silverstone A.E., Rosenbaum P.F., Weinstock R.S., Bartell S.M., Foushee H.R., Shelton C., Pavuk M. (2012). Polychlorinated Biphenyl (PCB) Exposure and Diabetes: Results from the Anniston Community Health Survey. Environ. Health Perspect..

[44] Wang S.L., Tsai P.C., Yang C.Y., Leon Guo Y. (2008). Increased Risk of Diabetes and Polychlorinated Biphenyls and Dioxins: A 24-year follow-up study of the Yucheng cohort. Diabetes Care.

[45] Reale C., Russo F., Credendino S., Cuomo D., De Vita G., Mallardo M., Pennino F., Porreca I., Triassi M., De Felice M. (2019). A Toxicogenomic Approach Reveals a Novel Gene Regulatory Network Active in In Vitro and In Vivo Models of Thyroid Carcinogenesis. Int. J. Environ. Res. Public Health.

[46] Lans M.C., Spiertz C., Brouwer A., Koeman J.H. (1994). Different competition of thyroxine binding to transthyretin and thyroxine-binding globulin by hydroxy-PCBs, PCDDs and PCDFs. Eur. J. Pharmacol. Environ. Toxicol. Pharmacol..

[47] Brouwer A., Morse D.C., Lans M.C., Gerlienke Schuur A., Murk A.J., Klasson-Wehler E., Bergman Å., Visser T.J. (1998). Interactions of Persistent Environmental Organohalogens With the Thyroid Hormone System: Mechanisms and Possible Consequences for Animal and Human Health. Toxicol. Ind. Health.

[48] Pavuk M., Schecter A.J., Akhtar F.Z., Michalek J.E. (2003). Serum 2,3,7,8-Tetrachlorodibenzo-p-dioxin (TCDD) Levels and Thyroid Function in Air Force Veterans of the Vietnam War. Ann. Epidemiol..

[49] Haymart M.R., Repplinger D.J., Leverson G.E., Elson D.F., Sippel R.S., Jaume J.C., Chen H. (2008). Higher Serum Thyroid Stimulating Hormone Level in Thyroid Nodule Patients Is Associated with Greater Risks of Differentiated Thyroid Cancer and Advanced Tumor Stage. J. Clin. Endocrinol. Metab..

[50] Kim H.K., Yoon J.H., Kim S.J., Cho J.S., Kweon S.-S., Kang H.-C. (2012). Higher TSH level is a risk factor for differentiated thyroid cancer. Clin. Endocrinol..

[51] Kerger B.D., Scott P.K., Pavuk M., Gough M., Paustenbach D.J. (2012). Re-analysis of Ranch Hand study supports reverse causation hypothesis between dioxin and diabetes. Crit. Rev. Toxicol..

[52] Safe S.H. (1998). Development validation and problems with the toxic equivalency factor approach for risk assessment of dioxins and related compounds. J. Anim. Sci..

[53] Ceriello A. (2008). Possible Role of Oxidative Stress in the Pathogenesis of Hypertension. Diabetes Care.

[54] Wang S.-L., Chang Y.-C., Chao H.-R., Li C.-M., Li L.-A., Lin L.-Y., Päpke O. (2006). Body Burdens of Polychlorinated Dibenzo- p -dioxins, Dibenzofurans, and Biphenyls and Their Relations to Estrogen Metabolism in Pregnant Women. Environ. Health Perspect..

[55] Michalek J.E., Pavuk M. (2008). Diabetes and Cancer in Veterans of Operation Ranch Hand After Adjustment for Calendar Period, Days of Spraying, and Time Spent in Southeast Asia. J. Occup. Environ. Med..

[56] Bichteler A., Wikoff D.S., Loko F., Harris M.A. (2017). Estimating serum concentrations of dioxin-like compounds in the US population effective 2005–2006 and 2007–2008: A multiple imputation and trending approach incorporating NHANES pooled sample data. Environ. Int..

