# Supplementary material for: The Association between Blood Concentrations of PCDD/DFs, DL-PCBs and the Risk of Type 2 Diabetes Mellitus and Thyroid Cancer in South Korea
Source: Int J Environ Res Public Health. 2022 Jul 18;19(14):8745. doi: 10.3390/ijerph19148745 (PMC9320419; doi:10.3390/ijerph19148745)
Supplement: Supplementary file 1 [file ijerph-19-08745-s001.zip › ijerph-1770965-supplementary.pdf]

The Association between Blood Concentrations of PCDD/DFs, DL-PCBs and the Risk of Type 2 Diabetes Mellitus and Thyroid Cancer in South Korea

SuHyun Lee, YoungWook Lim, YounSeok Kang, KeumJi Jung and SunHa Jee

Supplementary Table Legends

Supplementary Table S1. Serum concentrations for homologue of PCDD/DFs, DL-PCBs of the study group.

Supplementary Table S2. Odds Ratios of blood concentrations for homologue of PCDD/DFs, DL-PCBs (per 1 SD) and Type 2 Diabetes mellitus.

Supplementary Table S3. Odds Ratios of blood concentrations for homologue of PCDD/DFs, DL-PCBs (per 1 SD) and Thyroid Cancer.

Supplementary Figure Legends

Supplementary Figure S1. Flow Chart: Sample for the Analysis, KCPS-II.

Supplementary Figure S2. Schematic diagram showing different groups of blood samples tested for blood concentrations of PCDD/DFs, DL-PCBs (pgTEQ/g_lipid).

Supplementary Table S1. Serum concentrations for homologue of PCDD/DFs, DL-PCBs of the study group.

Congener	Mean(SD)				*p*-Value
	Overall	Control (n=55)	Type 2 Diabetes (n=30)	Thyroid Cancer (n=15)	
**PCDDs (pg/g-lipid)**					
**2378-TCDD**	0.46(1.16)	0.43(1.34)	0.27(0.59)	0.97(1.24)	0.1546
**12378-PeCDD**	1.98(5.2)	0.20(1.49)	2.59(4.96)	7.25(9.43)	<0.0001
**123478-HxCDD**	0.80(2.15)	0.50(1.98)	1.06(2.24)	1.38(2.53)	0.2792
**123678-HxCDD**	5.36(6.2)	4.77(6.94)	6.27(4.7)	5.72(6.06)	0.5540
**123789-HxCDD**	2.1(4.54)	1.39(4.45)	3.08(4.71)	2.73(4.35)	0.2204
**1234678-HpCDD**	19.92(11.98)	16.86(13.74)	21.54(8.3)	27.88(5.72)	0.0038
**OCDD**	175.66(120.92)	187.58(147.08)	135.01(58.19)	213.26(85.58)	0.0667
**PCDFs (pg/g-lipid)**					
**2378-TCDF**	0.47(0.96)	0.24(0.8)	0.68(1.06)	0.92(1.11)	0.0168
**12378-PeCDF**	1.48(2.61)	1.42(2.91)	1.3(2.18)	2.03(2.31)	0.6638
**23478-PeCDF**	5.15(5.36)	4.96(5.94)	5.04(4.35)	6.07(5.16)	0.7743
**123478-HxCDF**	0.91(3.61)	1.24(4.2)	0.78(3.3)	0(0)	0.4892
**123678-HxCDF**	1.92(5.49)	1.09(4.69)	2.3(4.89)	4.21(8.37)	0.1337
**234678-HxCDF**	0.21(1.61)	0.38(2.16)	0(0)	0(0)	0.5024
**123789-HxCDF**	1.56(5.71)	1.79(6.34)	0.71(3.87)	2.39(6.46)	0.5871
**1234678-HpCDF**	7.93(5.93)	7.39(6.89)	8.52(4.15)	8.70(5.26)	0.6101
**1234789-HpCDF**	1.04(3.45)	0.81(3.44)	1.64(3.8)	0.71(2.76)	0.5310
**OCDF**	2.69(6.36)	2.93(7.15)	1.98(5.19)	3.21(5.58)	0.7589
**Non-ortho PCBs (pg/g-lipid)**					
**4CB-77**	20.1(21.13)	14.83(18.04)	27.76(27.78)	24.14(7.26)	0.0176

**4CB-81**	0.18(0.8)	0(0)	0.31(1.01)	0.55(1.47)	0.0346
**5CB-126**	27.05(13.66)	25.14(15.17)	28.99(10.36)	30.13(13.34)	0.2983
**6CB-169**	16.85(11.56)	14.92(12.81)	18.72(9.25)	20.2(10.07)	0.1687
**Mono-ortho PCBs (pg/g-lipid)**					
**5CB-105**	1398.23(700.32)	1379.68(637.16)	1357.08(499.87)	1548.52(1164.26)	0.6638
**5CB-114**	257.75(122.06)	276.16(136.34)	230.75(81.2)	244.26(130.04)	0.2362
**5CB-118**	6619.94(3294.99)	6450.87(2957.3)	6481.58(2334.79)	7516.62(5543.86)	0.5245
**5CB-123**	104.37(50.78)	96.59(46.39)	109.93(42.46)	121.80(74.63)	0.1821
**6CB-156**	2039.34(1034.15)	2121.21(1092.27)	1909.91(833.27)	1998.04(1207.69)	0.6620
**6CB-157**	583.97(281.45)	586.22(274.94)	558.15(239.89)	627.33(381.99)	0.7403
**6CB-167**	1026.99(562.15)	980.54(472.17)	1000.27(419.33)	1250.74(973.04)	0.2462
**7CB-189**	282.71(157.16)	288.68(160.78)	274.87(141.31)	276.48(182.41)	0.9166

SD, standard deviation

Supplementary Table S2. Odds Ratios of blood concentrations for homologue of PCDD/DFs, DL-PCBs (per 1 SD) and Type 2 Diabetes mellitus.

Exposure	Dataset	Model 1	Model 2
		OR (95% CI)	
**PCDDs**			
**2378_TCDD**	Total	0.84 (0.50–1.39)	0.82 (0.42–1.59)
	Male	0.85 (0.41–1.76)	0.32 (0.10–1.01)
	Female	0.81 (0.39–1.66)	1.32 (0.57–3.07)
**12378_PeCDD**	Total	*3.38 (1.27–9.00)*	*4.21 (1.09–16.30)*
	Male	1.64 (0.67–4.04)	1.25 (0.47–3.35)
	Female	-	-
**123478_HxCDD**	Total	1.29 (0.83–2.01)	1.55 (0.93–2.57)
	Male	0.70 (0.25–1.96)	0.88 (0.27–2.87)
	Female	1.8 (0.92–3.51)	2.32 (0.96–5.60)
**123678_HxCDD**	Total	1.29 (0.82–2.03)	1.31 (0.79–2.17)
	Male	1.39 (0.72–2.67)	1.47 (0.68–3.20)
	Female	1.25 (0.66–2.37)	1.26 (0.62–2.54)
**123789_HxCDD**	Total	1.45 (0.90–2.34)	1.52 (0.90–2.57)
	Male	1.05 (0.59–1.90)	0.35 (0.11–1.14)
	Female	*3.48 (1.04–11.63)*	*3.89 (1.04–14.60)*
**1234678_HpCDD**	Total	1.46 (0.92–2.34)	1.61 (0.94–2.77)
	Male	1.55 (0.85–2.84)	2.09 (0.92–4.74)
	Female	1.63 (0.67–3.96)	1.50 (0.54–4.18)

**OCDD**	Total	0.46 (0.21–1.01)	*0.34 (0.14–0.85)*
	Male	*0.07 (0.01–0.74)*	*0.03 (0.00–0.51)*
	Female	0.91 (0.38–2.14)	0.64 (0.24–1.66)
**PCDFs**			
**2378-TCDF**	Total	*1.61 (1.01–2.57)*	*1.71 (1.00–2.91)*
	Male	1.50 (0.84–2.67)	1.70 (0.76–3.78)
	Female	2.29 (0.95–5.51)	2.6 (0.82–8.23)
**12378-PeCDF**	Total	0.95 (0.60–1.50)	1.03 (0.61–1.72)
	Male	0.91 (0.45–1.85)	0.93 (0.39–2.23)
	Female	0.97 (0.53–1.78)	1.05 (0.52–2.13)
**23478-PeCDF**	Total	1.00 (0.64–1.58)	1.38 (0.81–2.38)
	Male	1.08 (0.53–2.21)	1.90 (0.68–5.35)
	Female	1 (0.54–1.85)	1.12 (0.55–2.28)
**123478-HxCDF**	Total	0.89 (0.53–1.48)	0.74 (0.42–1.30)
	Male	-	-
	Female	0.90 (0.54–1.52)	0.71 (0.39–1.29)
**123678-HxCDF**	Total	1.34 (0.84–2.14)	1.34 (0.82–2.19)
	Male	-	-
	Female	1.18 (0.71–1.97)	1.09 (0.64–1.85)
**234678-HxCDF**	Total	-	-
	Male	-	-
	Female	-	-
**123789-HxCDF**	Total	0.79 (0.43–1.44)	0.97 (0.51–1.82)
	Male	1.05 (0.57–1.92)	1.33 (0.70–2.52)

	Female	-	-
**1234678-HpCDF**	Total	1.21 (0.77–1.90)	1.33 (0.78–2.27)
	Male	0.67 (0.34–1.34)	0.48 (0.18–1.28)
	Female	*4.20 (1.51–11.66)*	*6.56 (1.59–27.01)*
**1234789-HpCDF**	Total	1.34 (0.83–2.14)	*1.93 (1.11–3.34)*
	Male	2.12 (0.54–8.27)	2.14 (0.47–9.76)
	Female	1.27 (0.77–2.08)	*2.09 (1.05–4.18)*
**OCDF**	Total	*1.61 (1.01–2.57)*	*1.71 (1.00–2.91)*
	Male	1.50 (0.84–2.67)	1.7 (0.76–3.78)
	Female	2.29 (0.95–5.51)	2.6 (0.82–8.23)
**Non-ortho PCBs**			
**4CB-77**	Total	0.86 (0.52–1.43)	1.09 (0.63–1.88)
	Male	1.41 (0.63–3.16)	1.88 (0.70–5.04)
	Female	0.61 (0.26–1.45)	0.67 (0.26–1.73)
**4CB-81**	Total	*1.82 (1.07–3.08)*	*1.81 (1.02–3.24)*
	Male	1.06 (0.59–1.92)	0.75 (0.36–1.57)
	Female	-	-
**5CB-126**	Total	-	-
	Male	-	-
	Female	-	-
**6CB-169**	Total	1.32 (0.83–2.10)	1.43 (0.85–2.41)
	Male	1.53 (0.82–2.84)	1.66 (0.79–3.49)
	Female	1.13 (0.53–2.40)	1.21 (0.52–2.81)
**Mono-ortho PCBs**			

**5CB-105**	Total	0.96 (0.61–1.52)	1.00 (0.60–1.66)
	Male	1.27 (0.70–2.33)	1.38 (0.68–2.80)
	Female	0.61 (0.28–1.36)	0.64 (0.26–1.58)
**5CB-114**	Total	0.63 (0.36–1.09)	0.70 (0.39–1.27)
	Male	1.00 (0.49–2.04)	0.98 (0.44–2.19)
	Female	*0.34 (0.12–0.98)*	0.40 (0.13–1.23)
**5CB-118**	Total	1.01 (0.64–1.59)	1.05 (0.63–1.75)
	Male	1.42 (0.77–2.61)	1.50 (0.73–3.04)
	Female	0.60 (0.27–1.33)	0.64 (0.25–1.62)
**5CB-123**	Total	1.34 (0.85–2.11)	1.44 (0.88–2.37)
	Male	1.49 (0.80–2.79)	1.65 (0.82–3.30)
	Female	1.20 (0.61–2.39)	1.43 (0.64–3.19)
**6CB-156**	Total	0.79 (0.48–1.28)	0.85 (0.50–1.46)
	Male	1.22 (0.66–2.26)	1.24 (0.61–2.50)
	Female	0.34 (0.12–1.01)	0.37 (0.11–1.22)
**6CB-157**	Total	0.89 (0.56–1.42)	1.00 (0.59–1.69)
	Male	1.64 (0.85–3.15)	1.72 (0.79–3.72)
	Female	0.35 (0.13–0.95)	0.39 (0.13–1.19)
**6CB-167**	Total	1.04 (0.66–1.65)	1.15 (0.68–1.93)
	Male	1.66 (0.88–3.13)	1.85 (0.88–3.89)
	Female	0.49 (0.19–1.24)	0.50 (0.17–1.48)
**7CB-189**	Total	0.90 (0.56–1.44)	0.94 (0.55–1.61)
	Male	1.62 (0.85–3.07)	1.73 (0.78–3.85)
	Female	0.30 (0.09–1.00)	0.28 (0.07–1.13)

OR, Odds ratio; CI, confidence interval.

Model 1: Adjusted for age and sex

Model 2: Adjusted for the model 1 variables, body mass index, systolic blood pressure and high-density lipoprotein

Marked in italics based on statistically significant results.

Supplementary Table S3. Odds Ratios of blood concentrations for homologue of PCDD/DFs, DL-PCBs (per 1 SD) and Thyroid Cancer.

Exposure	Dataset	Model 1	Model 2	Model 3
			OR (95% CI)	
**PCDDs**				
**2,3,7,8-TCDD**	Total	1.47 (0.86–2.49)	1.49 (0.87–2.56)	1.50 (0.87–2.58)
	Male	1.40 (0.64–3.07)	1.40 (0.64–3.06)	1.49 (0.65–3.38)
	Female	1.51 (0.73–3.13)	1.59 (0.75–3.39)	1.94 (0.80–4.72)
**1,2,3,7,8-PeCDD**	Total	*5.31 (1.72–16.32)*	*5.61 (1.80–17.46)*	*5.55 (1.77–17.39)*
	Male	*3.23 (1.10–9.48)*	*3.23 (1.11–9.35)*	*3.36 (1.12–10.09)*
	Female	-	-	-
**1,2,3,4,7,8-HxCDD**	Total	1.44 (0.87–2.38)	1.49 (0.88–2.51)	1.49 (0.88–2.53)
	Male	1.41 (0.68–2.92)	1.48 (0.69–3.16)	1.46 (0.68–3.14)
	Female	1.46 (0.73–2.92)	1.49 (0.72–3.08)	1.48 (0.69–3.19)
**1,2,3,6,7,8-HxCDD**	Total	1.17 (0.66–2.06)	1.17 (0.66–2.07)	1.17 (0.66–2.09)
	Male	1.38 (0.59–3.21)	1.36 (0.59–3.17)	1.46 (0.61–3.51)
	Female	0.98 (0.43–2.26)	0.79 (0.31–2.03)	0.79 (0.31–2.07)
**1,2,3,7,8,9-HxCDD**	Total	1.33 (0.80–2.20)	1.36 (0.81–2.28)	1.41 (0.83–2.40)
	Male	1.00 (0.48–2.07)	0.99 (0.48–2.06)	0.97 (0.46–2.04)
	Female	2.49 (0.86–7.21)	2.45 (0.80–7.53)	*3.10 (1.01–9.44)*
**1,2,3,4,6,7,8-HpCDD**	Total	*2.60 (1.29–5.21)*	*2.77 (1.34–5.72)*	*2.80 (1.35–5.79)*
	Male	1.72 (0.74–3.98)	1.69 (0.73–3.92)	1.72 (0.72–4.07)
	Female	*6.34 (1.60–25.19)*	*6.36 (1.56–25.98)*	*6.51 (1.56–27.18)*
**OCDD**	Total	1.21 (0.71–2.08)	1.24 (0.72–2.14)	1.24 (0.72–2.15)
	Male	0.82 (0.29–2.27)	0.82 (0.30–2.27)	0.85 (0.31–2.32)
	Female	2.01 (0.78–5.17)	1.86 (0.73–4.79)	1.86 (0.71–4.89)

**PCDFs**				
**2,3,7,8-TCDF**	Total	*2.01 (1.16–3.47)*	*2.16 (1.21–3.87)*	*2.17 (1.21–3.87)*
	Male	1.57 (0.75–3.30)	1.55 (0.72–3.32)	1.57 (0.72–3.44)
	Female	*2.96 (1.13–7.75)*	*4.22 (1.24–14.43)*	*4.75 (1.39–16.21)*
**1,2,3,7,8-PeCDF**	Total	1.24 (0.73–2.12)	1.23 (0.72–2.11)	1.23 (0.72–2.11)
	Male	2.22 (0.96–5.11)	2.19 (0.94–5.11)	*2.48 (1.01–6.11)*
	Female	0.73 (0.29–1.83)	0.70 (0.26–1.83)	0.69 (0.25–1.88)
**2,3,4,7,8-PeCDF**	Total	1.22 (0.68–2.17)	1.25 (0.69–2.24)	1.24 (0.69–2.24)
	Male	2.20 (0.76–6.38)	2.24 (0.73–6.81)	2.26 (0.73–7.01)
	Female	0.86 (0.39–1.87)	0.92 (0.42–2.02)	0.88 (0.39–1.98)
**1,2,3,4,7,8-HxCDF**	Total	-	-	-
	Male	-	-	-
	Female	-	-	-
**1,2,3,6,7,8-HxCDF**	Total	1.55 (0.92–2.61)	1.64 (0.96–2.80)	1.74 (0.99–3.07)
	Male	-	-	-
	Female	0.67 (0.18–2.47)	0.68 (0.17–2.77)	0.72 (0.17–3.04)
**2,3,4,6,7,8-HxCDF**	Total	-	-	-
	Male	-	-	-
	Female	-	-	-
**1,2,3,7,8,9-HxCDF**	Total	1.08 (0.62–1.87)	1.12 (0.64–1.94)	1.12 (0.64–1.95)
	Male	1.21 (0.54–2.71)	1.22 (0.55–2.74)	1.24 (0.55–2.80)
	Female	0.97 (0.46–2.06)	0.97 (0.45–2.08)	0.98 (0.45–2.10)
**1,2,3,4,6,7,8-HpCDF**	Total	1.34 (0.73–2.46)	1.49 (0.79–2.81)	1.52 (0.80–2.89)
	Male	1.13 (0.45–2.82)	1.11 (0.45–2.74)	1.10 (0.45–2.67)
	Female	1.78 (0.63–4.99)	2.00 (0.67–5.98)	2.09 (0.69–6.33)
**1,2,3,4,7,8,9-HpCDF**	Total	0.93 (0.50–1.75)	0.93 (0.49–1.76)	0.93 (0.49–1.77)

	Male	-	-	-
	Female	0.96 (0.52–1.80)	0.91 (0.48–1.75)	0.93 (0.48–1.80)
**OCDF**	Total	1.02 (0.57–1.81)	1.04 (0.58–1.87)	1.04 (0.58–1.87)
	Male	2.43 (0.92–6.40)	2.44 (0.92–6.46)	*3.06 (1.02–9.16)*
	Female	0.48 (0.13–1.72)	0.41 (0.10–1.63)	0.39 (0.10–1.61)
**Non-ortho PCBs**				
**4CB-77**	Total	*1.93 (1.03–3.60)*	*1.93 (1.02–3.64)*	*1.92 (1.02–3.64)*
	Male	1.18 (0.61–2.26)	1.17 (0.61–2.26)	1.21 (0.62–2.38)
	Female	-	-	-
**4CB-81**	Total	-	-	-
	Male	-	-	-
	Female	-	-	-
**5CB-126**	Total	1.43 (0.80–2.58)	1.44 (0.80–2.60)	1.44 (0.80–2.61)
	Male	1.56 (0.72–3.37)	1.55 (0.68–3.50)	1.60 (0.70–3.66)
	Female	1.26 (0.47–3.36)	1.21 (0.43–3.39)	1.22 (0.42–3.56)
**6CB-169**	Total	1.59 (0.87–2.91)	1.62 (0.88–2.98)	1.61 (0.88–2.98)
	Male	2.01 (0.84–4.81)	2.01 (0.84–4.82)	2.05 (0.84–4.99)
	Female	1.23 (0.51–2.97)	1.18 (0.48–2.93)	1.10 (0.43–2.83)
**Mono-ortho PCBs**				
**5CB-105**	Total	1.27 (0.74–2.20)	1.25 (0.71–2.18)	1.24 (0.71–2.18)
	Male	1.65 (0.81–3.36)	1.68 (0.79–3.57)	1.67 (0.78–3.57)
	Female	0.71 (0.25–2.04)	0.60 (0.19–1.90)	0.52 (0.16–1.73)
**5CB-114**	Total	0.76 (0.40–1.47)	0.75 (0.39–1.46)	0.75 (0.38–1.46)
	Male	1.03 (0.41–2.57)	0.97 (0.37–2.56)	0.99 (0.37–2.62)
	Female	0.58 (0.21–1.56)	0.51 (0.17–1.53)	0.44 (0.13–1.44)
**5CB-118**	Total	1.36 (0.79–2.34)	1.33 (0.77–2.31)	1.33 (0.77–2.31)

	Male	1.62 (0.81–3.25)	1.65 (0.78–3.47)	1.65 (0.78–3.49)
	Female	0.91 (0.33–2.50)	0.82 (0.28–2.40)	0.69 (0.22–2.18)
**5CB-123**	Total	1.63 (0.93–2.86)	1.61 (0.92–2.84)	1.61 (0.92–2.84)
	Male	2.28 (0.93–5.59)	2.36 (0.95–5.86)	2.38 (0.94–6.04)
	Female	1.08 (0.43–2.68)	0.97 (0.37–2.55)	0.89 (0.33–2.44)
**6CB-156**	Total	0.91 (0.49–1.69)	0.89 (0.48–1.67)	0.89 (0.48–1.66)
	Male	1.26 (0.60–2.65)	1.22 (0.56–2.67)	1.23 (0.56–2.73)
	Female	0.54 (0.18–1.61)	0.48 (0.14–1.57)	0.39 (0.11–1.43)
**6CB-157**	Total	1.17 (0.67–2.05)	1.17 (0.66–2.05)	1.16 (0.66–2.04)
	Male	1.59 (0.77–3.26)	1.58 (0.75–3.35)	1.60 (0.75–3.42)
	Female	0.71 (0.27–1.84)	0.66 (0.24–1.82)	0.55 (0.18–1.67)
**6CB-167**	Total	1.55 (0.89–2.71)	1.55 (0.88–2.70)	1.54 (0.88–2.70)
	Male	1.75 (0.85–3.63)	1.79 (0.84–3.83)	1.79 (0.83–3.88)
	Female	1.18 (0.41–3.39)	1.12 (0.37–3.38)	0.99 (0.31–3.17)
**7CB-189**	Total	0.95 (0.52–1.73)	0.92 (0.50–1.71)	0.92 (0.50–1.70)
	Male	1.53 (0.72–3.25)	1.51 (0.69–3.31)	1.51 (0.68–3.36)
	Female	0.45 (0.14–1.45)	0.38 (0.10–1.40)	0.32 (0.08–1.31)

OR, Odds ratio; CI, confidence interval.

Model 1: Adjusted for age and sex

Model 2: Adjusted for the model 1 variables and body mass index

Model 3: Adjusted for the model 2 variables and thyroid stimulating hormone

Marked in italics based on statistically significant results.

Supplementary Figure S1. Flow Chart: Sample for the Analysis, KCPS-II.

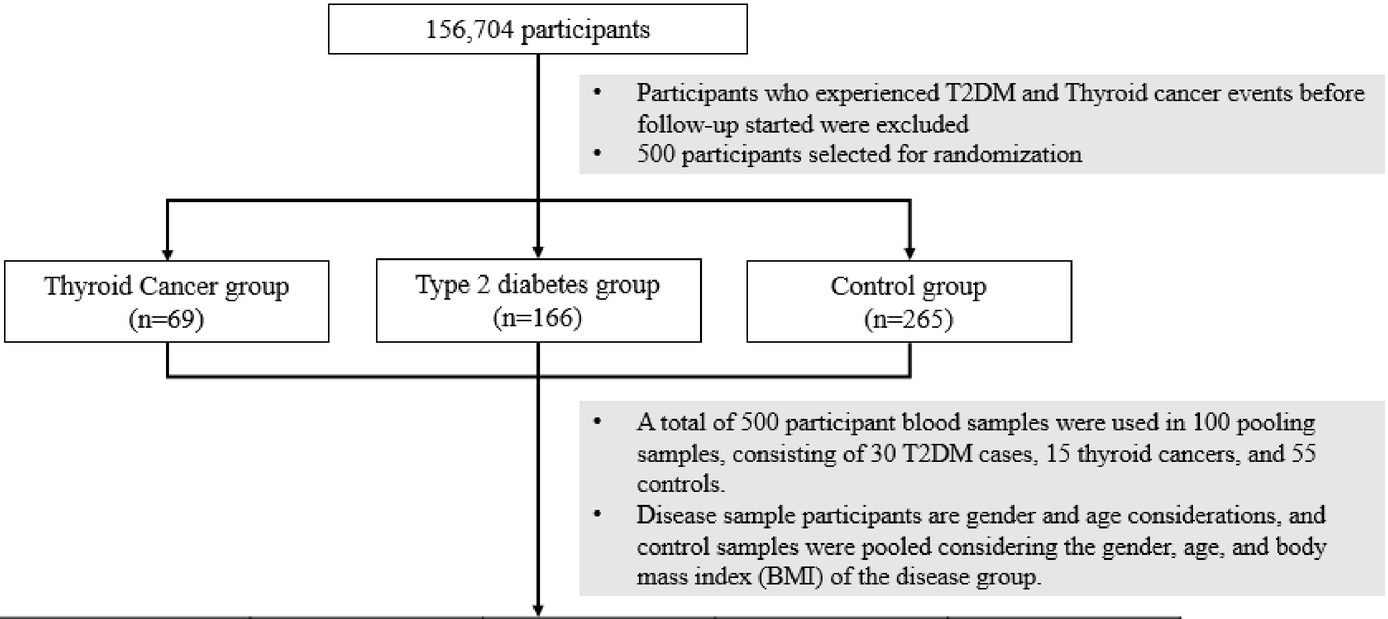

			Thyroid Cancer	Type 2 diabetes	Control	Overall
			N (number of Pooled sample)			
Overall			15 (69)	30 (166)	55 (265)	100 (500)
Men	Age group	<50	2 (10)	5 (37)	9 (46)	16 (93)
		50-59	2 (8)	5 (33)	9 (46)	16 (87)
		60≤	2 (10)	5 (28)	9 (45)	16 (83)
Women	Age group	<50	3 (13)	5 (23)	10 (47)	18 (83)
		50-59	3 (14)	5 (23)	9 (40)	17 (77)
		60≤	3 (14)	5 (22)	9 (41)	17 (77)

Supplementary Figure S2. Schematic diagram showing different groups of blood samples tested for blood concentrations of PCDD/DFs, DL-PCBs (pgTEQ/g_lipid).

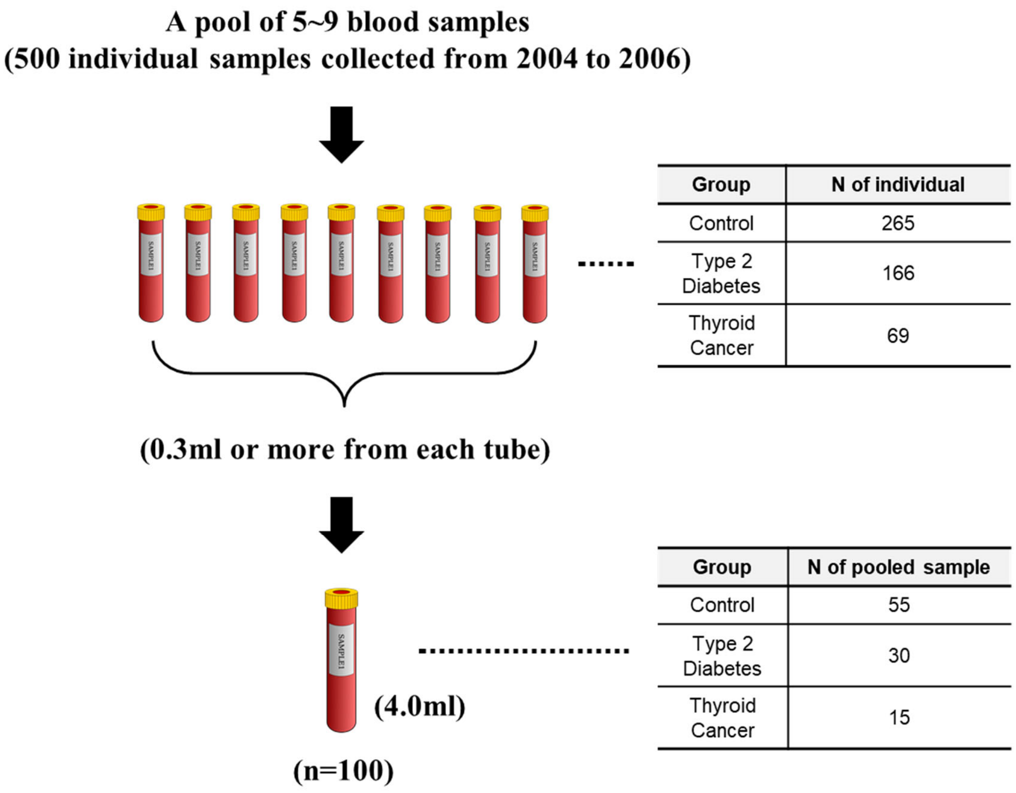

Group	N of individual
Control	265
Type 2 Diabetes	166
Thyroid Cancer	69

Group	N of pooled sample
Control	55
Type 2 Diabetes	30
Thyroid Cancer	15
